# Different RNA splicing mechanisms contribute to diverse infective outcome of classical swine fever viruses of differing virulence: insights from the deep sequencing data in swine umbilical vein endothelial cells

**DOI:** 10.7717/peerj.2113

**Published:** 2016-06-08

**Authors:** Pengbo Ning, Yulu Zhou, Wulong Liang, Yanming Zhang

**Affiliations:** 1 College of Veterinary Medicine, Northwest A&F University, Yangling, Shaanxi; 2 School of Life Science and Technology, Xidian University, Xi’an, China; 3 College of Science, Northwest A&F University, Yangling, China

**Keywords:** CSFV Shimen, RNA splicing, SUVEC, mTOR, CSFV C

## Abstract

Molecular mechanisms underlying RNA splicing regulation in response to viral infection are poorly understood. Classical swine fever (CSF), one of the most economically important and highly contagious swine diseases worldwide, is caused by classical swine fever virus (CSFV). Here, we used high-throughput sequencing to obtain the digital gene expression (DGE) profile in swine umbilical vein endothelial cells (SUVEC) to identify different response genes for CSFV by using both Shimen and C strains. The numbers of clean tags obtained from the libraries of the control and both CSFV-infected libraries were 3,473,370, 3,498,355, and 3,327,493 respectively. In the comparison among the control, CSFV-C, and CSFV-Shimen groups, 644, 158, and 677 differentially expressed genes (DEGs) were confirmed in the three groups. Pathway enrichment analysis showed that many of these DEGs were enriched in spliceosome, ribosome, proteasome, ubiquitin-mediated proteolysis, cell cycle, focal adhesion, Wnt signalling pathway, etc., where the processes differ between CSFV strains of differing virulence. To further elucidate important mechanisms related to the differential infection by the CSFV Shimen and C strains, we identified four possible profiles to assess the significantly expressed genes only by CSFV Shimen or CSFV C strain. GO analysis showed that infection with CSFV Shimen and C strains disturbed ‘RNA splicing’ of SUVEC, resulting in differential ‘gene expression’ in SUVEC. Mammalian target of rapamycin (mTOR) was identified as a significant response regulator contributed to impact on SUVEC function for CSFV Shimen. This computational study suggests that CSFV of differing virulence could induce alterations in RNA splicing regulation in the host cell to change cell metabolism, resulting in acute haemorrhage and pathological damage or infectious tolerance.

## Introduction

Classical swine fever virus (CSFV) is the etiological agent of classical swine fever (CSF), one of the most economically important and highly contagious swine disease worldwide (Tamura et al., 2014). Currently, the molecular mechanism underlying the pathogenesis of acute CSF is a key issue. Acute CSF is caused by the virulent strain of CSFV and shows a typical pathology, including haemorrhagic lymphadenitis in the lymph nodes and diffuse haemorrhage in the skin, kidney, and other organs (Moennig, Floegel-Niesmann & Greiser-Wilke, 2003). In addition, there are other symptoms such as high fever and depression (Moennig, Floegel-Niesmann & Greiser-Wilke, 2003).

Although CSFV does not cause cytopathic effects in host cells after infection, acute CSF is the cause of high mortality in pigs. The changes occurring in CSFV-infected host cells that could be involved in the pathogenesis of acute CSF are not well known. A few reports have tried to address this question by studying the response of host cells infected with CSFV (Durand et al., 2009; Li et al., 2010; Shi et al., 2009). These previous studies have demonstrated that the physiological function and intracellular environment of infected host cells undergo substantial changes because of CSFV-host interaction (Gladue et al., 2010; Hulst, Loeffen & Weesendorp, 2013). However, the previous research is mostly confined to cytokine-related changes upon CSFV infection (Zaffuto et al., 2007). A systematic study regarding the mechanism of acute infection by comparing classical swine fever viruses of differing virulence has not been conducted. It would be more interesting to study the changes that result in the pathological response triggered by CSFV than to study the general cell response elicited by CSFV as a xenobiotic, as the latter may be of little consequence to understanding the underlying pathological mechanisms.

For this purpose, we performed digital gene expression (DGE) tag profiling (Audic & Claverie, 1997), a high-throughput deep-sequencing method, to analysis the SUVEC transcriptome response to CSFV infection by using the Illumina Genome Analyser platform. In particular, infection with the CSFV C strain was used to identify differentially expressed genes in SUVEC compared with those observed in cells infected with the virulent CSFV Shimen strain. CSFV Shimen is a virulent strain that causes typical diffuse haemorrhage symptoms (Moennig, Floegel-Niesmann & Greiser-Wilke, 2003), whereas CSFV C completes its infection cycle without any pathological symptoms (Edwards et al., 2000). Thus, our study uncovered valuable findings and threw new light on the molecular interactions between CSFV and its host cells. Our results indicate that the host transcriptome undergoes considerable changes in response to CSFV infection of differing virulence, where CSFV Shimen and C strains implemented different mechanisms to disrupt the splicing regulation of host genes and induced changes in the differentiation and metabolic characteristics of the host cells, thereby resulting in different infection outcomes.

## Materials and Methods

### Culture, CSFV infection, and RNA isolation from SUVEC

The cell line derived from the immortalized SUVEC was obtained as previously described (Hong et al., 2007). The CSFV Shimen and C strains used in this study were obtained from the Control Institute of Veterinary Bioproducts and Pharmaceuticals (Beijing, China). SUVEC were cultured in 25-cm^2^ tissue culture flasks, at a density of 2 × 10^7^ cells per flask for further use. When SUVEC were 70–80% confluent, CSFV Shimen and C strains were added to respective cultures at a multiplicity of infection (MOI) of 10 (Ning et al., 2014). After 1 h of incubation at 37 °C in an atmosphere containing 5% CO_2_, the medium was aspirated and fresh medium containing 2% foetal calf serum was added, which was followed by incubation for 72 h in an atmosphere containing 5% CO_2_. High-resolution melt curve analyses were conducted to identify infection with CSFV, and quantitative PCR (qPCR) was carried out to detect CSFV proliferation (Ning et al., 2013). Total RNA was isolated with TRIzol reagent (Invitrogen, Carlsbad, CA, USA) from the CSFV-infected SUVEC and control samples after 72 h of infection, according to the manufacturer’s protocol. RNA yields were determined by measuring the absorbance of samples at 260 nm by using Nanodrop (ND-2000). Agilent 2100 Bioanalyzer (Agilent Technologies) was used to evaluate RNA integrity. Three high-quality samples (CSFV-Shimen infection, CSFV-C infection, and control) were separately submitted to DGE profiling based on Solexa sequencing.

### Library construction and Solexa sequencing

Sequencing libraries were created with the Illumina Gene Expression Sample Prep Kit (San Diego, CA, USA) and the Illumina Sequencing Chip (Flowcell), according to the manufacturer’s protocol. In brief, 6 µg mRNA was purified from total RNA by using adsorption to oligo (dT) magnetic beads. mRNA bound to Oligo (dT) beads was then converted to cDNA through reverse transcription. The four base recognition enzyme Nla I was then used to digest this cDNA, followed by ligation with Illumina adaptor 1. Mme I was used to digest the cDNA at 17 bp downstream of CATG sites, which was followed by ligation with Illumina adaptor 2 at the 3′ end. Primer GX1 and Primer GX2 were added for PCR. Then, 95-bp fragments were isolated by 60 g/L TBE PAGE. The DNA was purified and analysed by Illumina sequencing, with Illumina Cluster Station and Illumina HiSeq 2000 System used as the main instruments.

### Digital gene expression tag profiling and sequence annotation

Raw sequence reads were filtered through the Illumina pipeline, in which clean tags were obtained after filtering the adaptor tags and excluding low-quality tags and tags with a copy number of 1. The clean tags generated were mapped to the reference sequences in the UniGene database of *Sus scrofa* from the NCBI site (Bauer et al., 2010). Only the tags with a perfect match or one mismatch were accepted for further annotation based on reference genes. To estimate the expression level of each gene, the frequency of clean tags was normalized to the number of transcripts per million clean tags (TPM). Using TPM to compare differential gene expression levels across samples is a standard method and is extensively used in DGE analysis (Morrissy et al., 2009).

### Screening of differentially expressed genes (DEGs)

The probability that one gene is equally expressed in two samples was demonstrated as previously described (Audic & Claverie, 1997). The false discovery rate (FDR) was taken to determine the threshold of the *p*-value in multiple tests and analyses (Benjamini et al., 2001). In this study, the significance of differences in gene expression was determined by the threshold FDR ≤0.001 and the absolute value of log2-ratio ≥1 (Benjamini & Hochberg, 1995).

### Gene ontology (GO) and pathway enrichment analysis for DEGs

In gene expression profiling analysis (Xu et al., 2013), GO functional enrichment analysis identified significantly enriched GO terms in DEGs compared to the genomic background (Gene Ontology Consortium, 2006). Pathway enrichment analysis identified significantly enriched metabolic pathways or signal transduction pathways in DEGs, in consultation with the Kyoto Encyclopaedia of Genes and Genomes (KEGG) (Kanehisa et al., 2008).

### Cluster analysis of DEGs and querying interactions in Cytoscape

Cluster and Java Treeview software were used to perform cluster analysis of gene expression patterns for analysing similar expression patterns of genes (Saldanha, 2004). The software Cytoscape (Shannon et al., 2003) was used to establish an interaction network by distributing nodes into differential layers according to significantly over-represented biological processes (Ideker & Sharan, 2008; Zheng & Wang, 2008).

### Quantitative reverse transcriptase polymerase chain reaction analysis

Quantitative reverse transcriptase polymerase chain reaction (qRT-PCR) was performed on a Bio-Rad iQ5 system with SYBR Premix Ex Taq II (TaKaRa), using the same RNA samples that were employed for the DGE experiments. cDNA was synthesized using the Transcriptor First Strand cDNA Synthesis Kit (TaKaRa, Dalian, China), according to the manufacturer’s instruction. Each reaction was performed in triplicate, after which the average threshold cycle (Ct) was calculated per sample. The 2${}^{-\mrm{\Delta }\mrm{\Delta }CT}$ method was used to calculate the relative expression levels among CSFV infection and control samples described above.

### Western blot analysis

Cells were collected with cold PBS at indicated time points, then treated with RIPA lysis buffer whichcontains 1 mM phenylmethyl sulfonylfluoride (PMSF) (Beyotime, Beijing, China) on ice for 30 min. Protein concentration was confirmed using BCA Protein Assay Reagent (CWBIO, Beijing, China). Equivalent amounts of protein samples were separated by 12% SDS–PAGE and target protein were transferred to PVDF membranes. Membranes were blocked with 5% skim milk and then incubated with primary antibodies over night at 4 °C, followed by HRP-conjugated secondary antibodies. Signals were visualized by enhanced chemiluminescence solution (Advansta, USA) and using GeneGnome XRQ Chemidoc System (Syngene, Cambridge, UK) to obtain images.

### Enzyme-linked immunoassay (ELISA)

Porcine phosphorylation mTOR enzyme (p-mTOR) ELISA kit to monitor the levels of p-mTOR activation was obtained from Shanghai QiaoDu Bio-Tech Co., Ltd. Procedures were performed strictly according to the manufacturer’s instructions. SUVEC samples were seeded in 96-well plates after CSFV infection with a MOI of 10 at the indicated time points and allowed to incubate at 37 °C for 30 min. After washing five times, biotinylated antibody (50 µL) was added each well and incubate at 37 °C for 60 min. Enzyme-labeled antibody (50 µL) was then added to wells and allowed to incubate at 37 °C for 30 min. Plates were washed and color developed using TMB solution; after 15 min the enzyme reaction was stopped by adding stop solution. The A450 was determined by using a microplate reader (Multiskan FC, Thermo).

### Statistical analysis

Data were shown as means ± SEM values of three independent experiments. Each experiment was carried out in triplicate. Statistical comparisons were analyzed by oneway analysis of variance (ANOVA) using SPSS 16.0 software (SPSS Inc., Chicago, IL). A level of *P* < 0.05 was considered significant.

## Results

### qRT-PCR test results of CSFV

**Figure 1:**
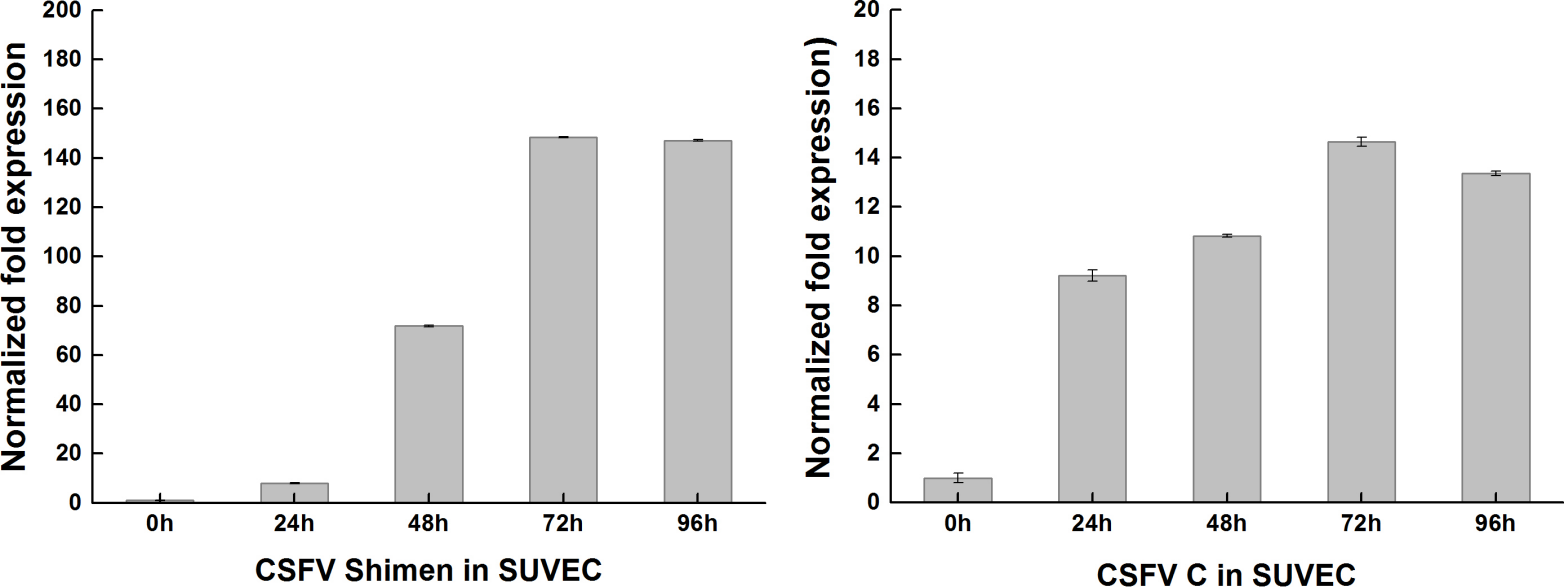
qRT-PCR results of CSFV Shimen and CSFV C strains after infection in SUVEC. qRT-PCR assays was performed to examine the expression of CSFV Shimen and CSFV C strains at 0, 24, 48, 72, 96h in SUVEC. *β*-actin was probed as the loading control.

SUVEC were inoculated with the CSFV Shimen strain or C strain and incubated for 0, 24, 48, and 72 h, followed by detection of CSFV replication by qPCR assay. As time progressed, Shimen and C strains of CSFV achieved exponential growth in SUVEC as shown in (Fig. 1). In addition, after culture for 72 h, the transcription of Shimen and C strains of CSFV attained peak values. By 96 h, the morphology of SUVEC was affected, owing to which, SUVEC could not correctly reflect virus proliferation and cytokine regulatory responses. This study therefore adopted cells infected by viruses for 72 h as samples for the DGE study.

### Analysis of DGE libraries and annotation of unique tags

In this study, global gene expression profiles in SUVEC were analysed using the Solexa/Illumina DGE system to investigate the molecular changes following infection with CSFV C and Shimen strains. cDNA libraries from uninfected and CSFV (C and Shimen)-infected SUVEC were sequenced using massive parallel sequencing, and the major characteristics of these three libraries were summarized in Table 1. A saturation analysis was performed to check whether the sequencing depth was sufficient for the transcriptome coverage. It is shown that the three libraries can be fully saturated with transcripts under different SUVEC samples (Fig. S1). The total number of sequenced tags obtained for the control and CSFV (C and Shimen)-infected SUVEC samples were 3,668,130, 3,655,051, and 3,512,523, respectively. After filtering the adaptor sequences and removing low-quality tags and tags with a copy number of 1, 3,473,370, 3,498,355, and 3,327,493 clean tags remained. We identified 129,644, 117,592, and 124,538 distinct clean tags from the control and CSFV (C and Shimen)-infected samples, respectively. The distribution of clean tag expression was used to evaluate the normality of the complete data. The clean tags were then mapped to the reference database as a primary step of annotation (Hegedüs et al., 2009). Considering the robustness of subsequent data analysis, we only used the tags that matched to one gene in each library for further analysis. In total, 14,037 (10.83% of distinct clean tags), 12,452 (10.59% of distinct clean tags), and 12,888 (10.35% of distinct clean tags) tags in the control and CSFV-infected libraries were mapped to the reference genes. Meanwhile, 11,515 (8.88%), 10,167 (8.65%), and 10,566 (8.48%) unambiguous tags were matched to the reference genes. Using the BLAST search engine (Schutte et al., 2002), the key gene products sharing high homology with *Sus scrofa* were obtained and are listed in Table S1.

**Table 1:** Summary statistics of tags in CSFV presence and control samples. Clean tags are the remaining tags after the filtering out of low-quality tags and tags with a copy number of 1 from the total raw data. Distinct clean tag is the unique mapping tags. All mapping represents the number of all tags mapped to the UniGene database; Unambiguous tags are the remaining clean tags after removal of the tags mapped to reference sequences from multiple genes.

Summary	Control	CSFV-C infection	CSFV-shimen infection
Total tag	3,668,130	3,655,051	3,512,523
Clean tag	3,473,370	3,498,355	3,327,493
Distinct clean tag	129,644	117,592	124,538
CopyNum ≥ 2	129,644	117,592	124,538
CopyNum > 5	52,588	44,490	49,488
CopyNum > 10	33,167	28,130	30,980
CopyNum > 20	20,056	17,492	18,716
CopyNum > 50	9,533	8,714	8,883
CopyNum > 100	4,968	4,759	4,666
Distinct tag number in all tag mapping to gene	14,037(10.83%)	12,452(10.59%)	12,888(10.35%)
Distinct tag number in unambiguous tag mapping to gene	11,515(8.88%)	10,167(8.65%)	10,566(8.48%)

### Identification of DEGs

To explore the dynamic gene expression changes in the CSFV-infected SUVEC, we identified DEGs from the normalized DGE data by pairwise comparison between the samples (CSFV-C vs. Control, CSFV-Shimen vs. Control, and CSFV-Shimen vs. CSFV-C). As shown in Fig. S2 with their clustered heat maps, overall gene expression patterns were clearly and sharply different among the control, CSFV-C, and CSFV-Shimen groups, and 644, 158, and 677 genes were confirmed as showing significantly different expression in the three compared groups (*p* < 0.00015, FDR < 0.001).

### Pathway enrichment analysis of DEGs for SPLICING

**Figure 2:**
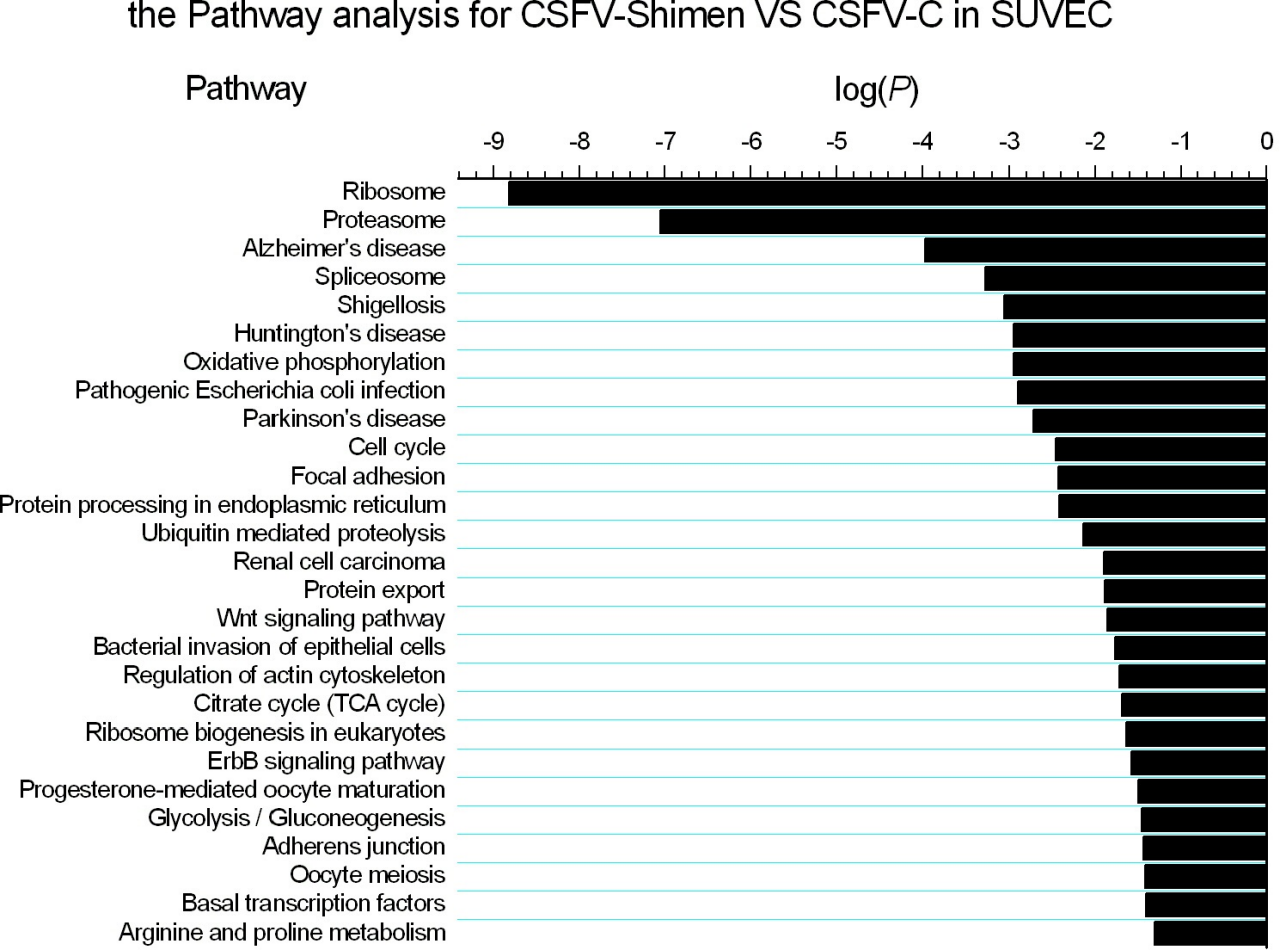
Pathway enrichment analysis for genes in CSFV Shimen-infected SUVEC vs. CSFV C-infected SUVEC. The vertical axis denotes the pathway category, and the horizontal axis denotes the negative log values (*p*-values) for the enriched terms.

**Figure 3:**
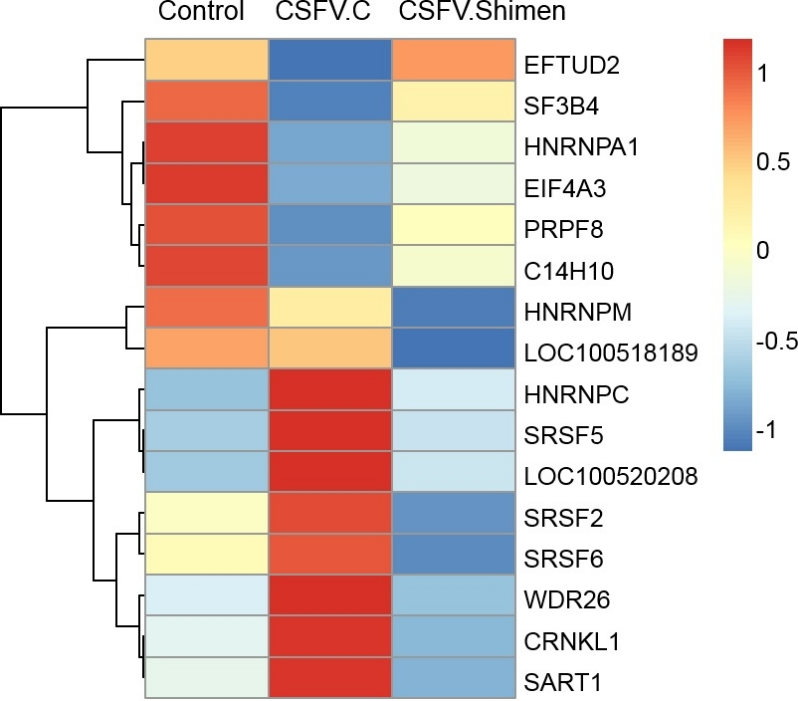
DGE of SUVECs reveals clusters of regulated genes enriched in spliceosome pathways that correlated with CSFV Shimen infection. The data obtained of CSFV-Shimen group were compared with those obtained from CSFV-C and Control (mock-infected cells) groups.

To characterize the functional roles of DEGs responsible for differences in CSFV infection, we performed pathway analysis of DEGs based on the KEGG database by using the two-sided Fisher’s exact test. Significant differences in signalling pathways were identified in SUVEC by pairwise comparison between the samples (CSFV C vs. Control and CSFV Shimen vs. Control respectively), in which the spliceosome pathway was significant as the most obvious pathway used by CSFV of differing virulence (Figs. S3 and S4). We further compared the difference in CSFV Shimen vs. CSFV C, and the significant signalling pathways included spliceosome, ribosome, proteasome, ubiquitin-mediated proteolysis, cell cycle, focal adhesion, and Wnt signalling pathways (Fig. 2). Key genes associated with spliceosomes show different responses to the C and Shimen strains of CSFV in Fig. 3.

### GO enrichment analysis for DEGs

**Figure 4:**
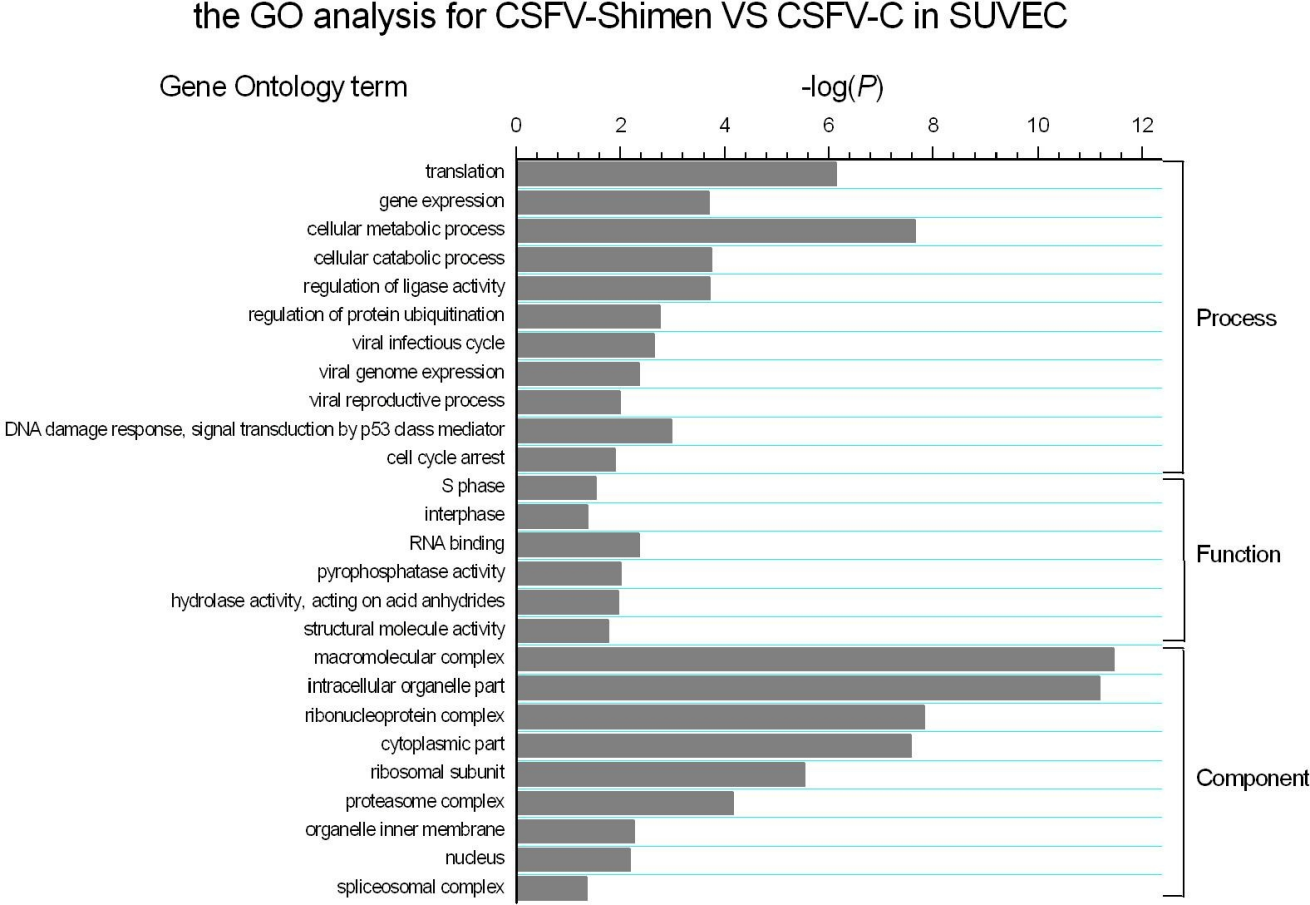
GO analysis for genes in CSFV Shimen-infected SUVEC vs. CSFV C-infected SUVEC. The vertical axis denotes the GO category, and the horizontal axis denotes negative log values (*p*-values) of the enriched terms.

To gain insight into the functional consequences of gene expression changes taking place in CSFV-infected SUVEC, we performed GO enrichment analysis of DEGs based on the GO database. Statistical significance was evaluated by two-sided Fisher’s exact test and *χ*^2^ test. We focused on GOs with *p* < 0.05 and FDR < 0.001. Analysis of GO distributions showed different GO distribution patterns in the GO process, function, and component between CSFV Shimen and CSFV C strains. As shown in Fig. 4, highly enriched GOs included gene expression, cellular metabolic process, viral infectious cycle, macromolecular complex, as well as spliceosomal complex.

### Trend analysis of DEGs

**Figure 5:**
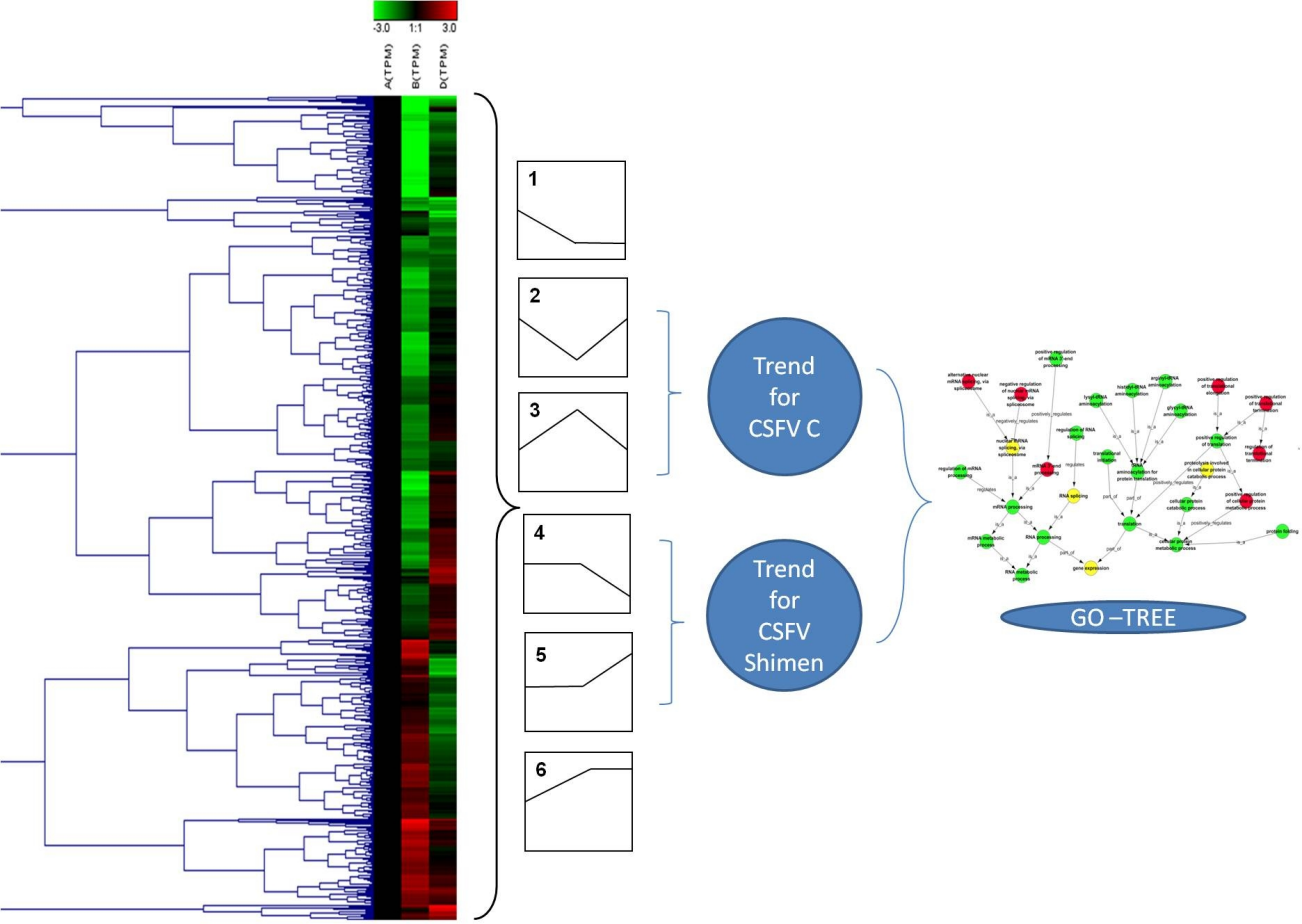
The procedure for series-cluster trend analysis to identify significant GO categories. Differentially expressed genes identified by pairwise comparisons among CSFV-Shimen, CSFV-C, and the control libraries are listed in 6 expression profiles. Profiles 2 and 3 indicate genes regulated by CSFV-C, and profiles 4 and 5 indicate genes regulated by CSFV-Shimen.

**Figure 6:**
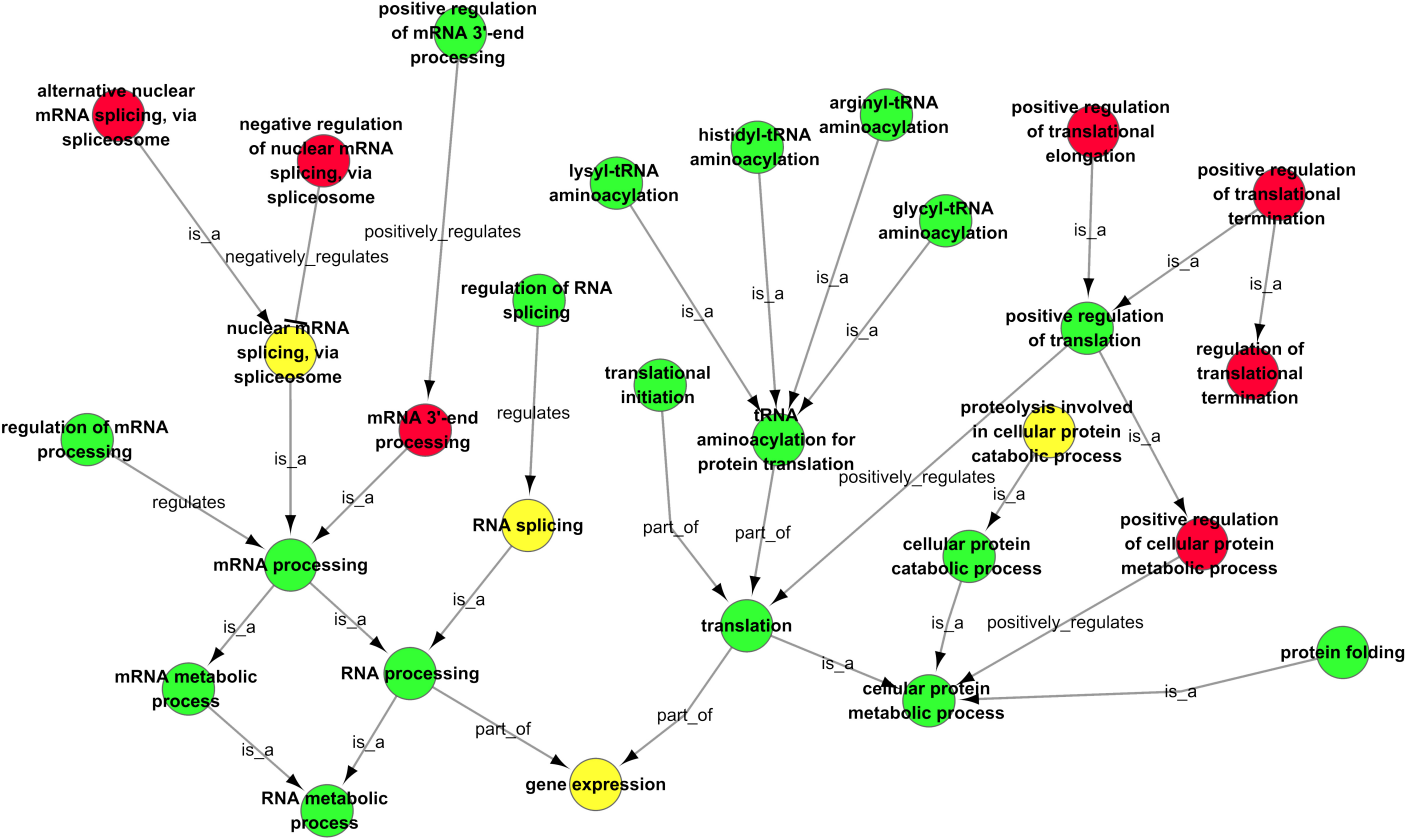
GO-Net of the significant GO categories in CSFV libraries vs. Control libraries (*p* < 0.05). Significant GO categories were identified by the procedure depicted in Fig. 6. Red dots represent the significant GO categories for infection with CSFV Shimen strain, green dots represent the significant GO categories for infection with CSFV C strain, and yellow dots represent the significant GO categories that responded to infection with both CSFV Shimen and CSFV C strains. The lines represent the interaction between GO categories.

We further analysed significantly differentially expressed genes only by CSFV Shimen or CSFV C strain. In subsequent Series-Cluster analysis with their clustered heat maps and hierarchical patterns, we identified six possible trends that represent the overall expression patterns (Fig. 5). Of these, profile 1 and profile 6 showed a sharp difference between the CSFV and Control groups. Specifically, we focused on profiles 2, 3, 4, and 5, in which SUVEC genes were down-regulated in profile 2 and up-regulated in profile 3 by CSFV C, and similarly, genes were down- or up regulated in profile 4 and profile 5 by CSFV Shimen strain alone. As shown with their GO analysis in Fig. 6, infection with CSFV Shimen and C strains disturbed ‘RNA splicing’ in SUVEC resulting in differential ‘gene expression’ in SUVEC, but the process differs between CSFV of differing virulence.

### Validation of DGE data by qRT-PCR

**Figure 7:**
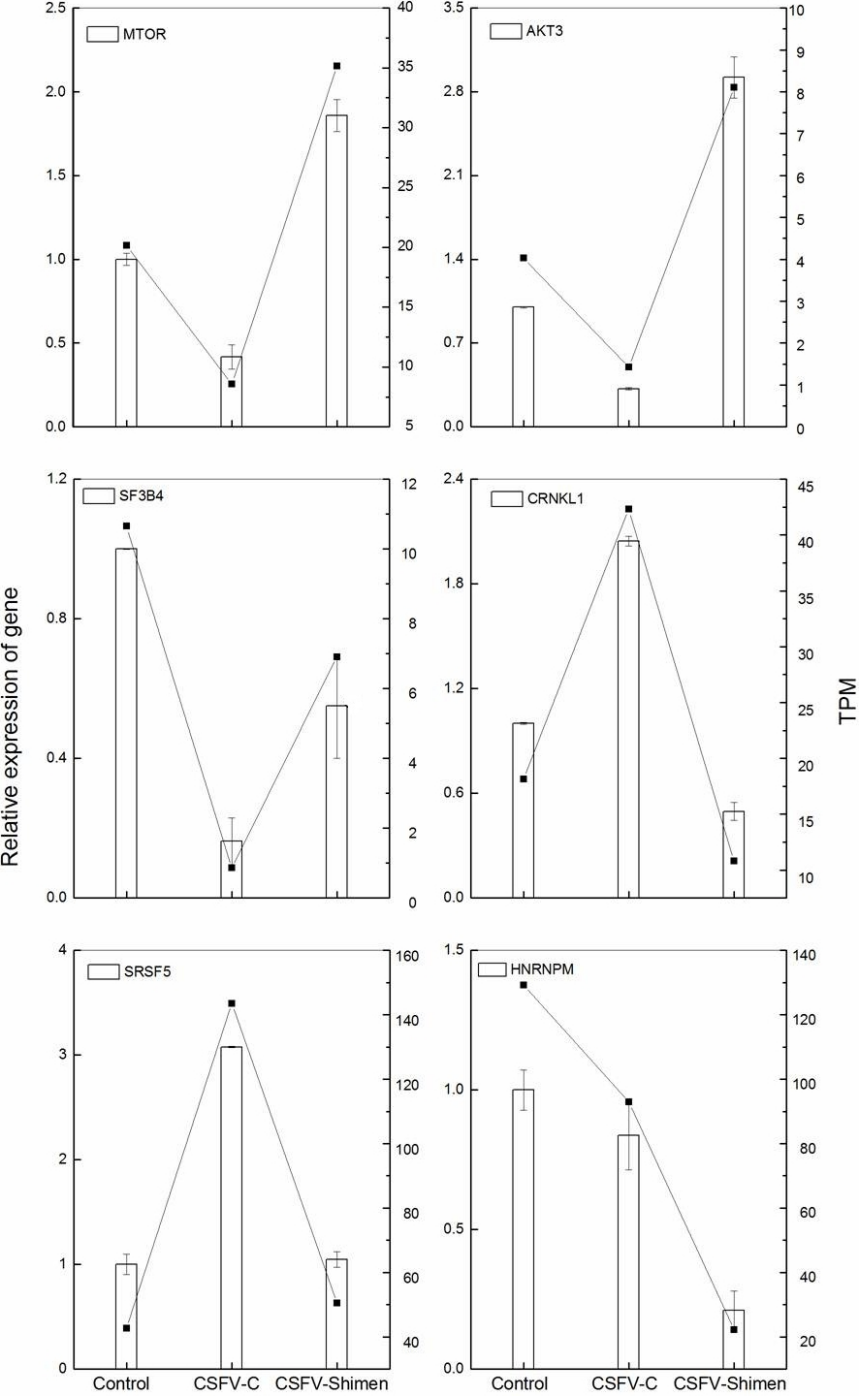
qPCR validation of the DGE data. The histograms denote the results of relative qPCR analysis of the target gene, and TPM changes are depicted in the line chart. TPM, transcripts per million mapped reads.

To validate the DGE results, qRT-PCR was conducted on six randomly selected SUVEC-responsive genes. qRT-PCR analysis results for genes agreed with the DGE data, indicating a good consistency between the qRT-PCR and DGE analysis (Fig. 7).

### Activation of mTOR during CSFV Shimen infection

**Figure 8:**
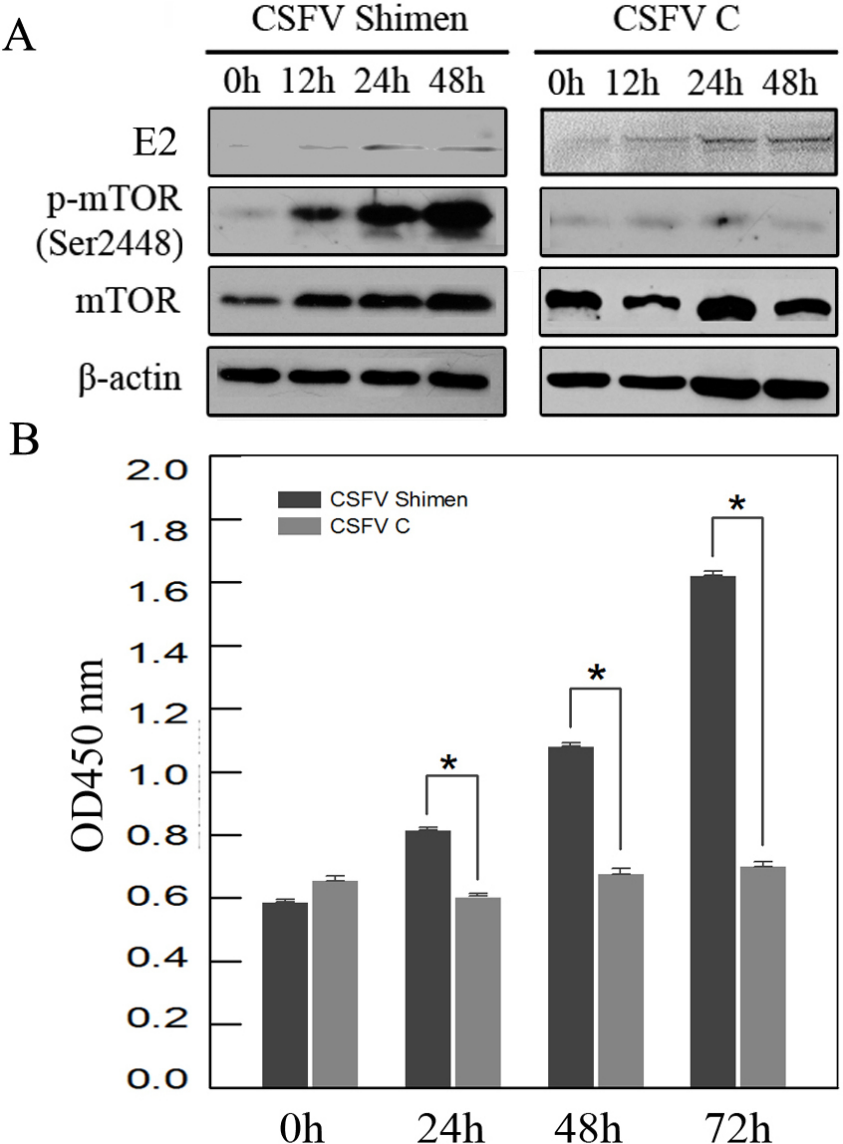
CSFV Shimen infection activates mTOR signalling pathways in SUVEC. (A) Whole-cell lysates from SUVEC after infection with CSFV Shimen strain were prepared and resolved by SDS-PAGE, transferred to nitrocellulose membranes, and immunoblotted. The protein levels of mTOR and its phosphorylated forms were analyzed. The amounts of *β*-actin were also assessed to monitor the equal loadings of protein extracts. (B) p-mTOR activation induced by CSFV Shimen infection was determined by using ELISA assay. These results are representative of three independent experiments. Values are means ± the SD from triplicate wells. p-, Phosphorylated.

DGE and qPCR analysis (Fig. S5, Fig. 7) suggest activation of mTOR would be involved in the infection of CSFV Shimen. In order to assess whether activation of mTOR signaling pathway occurred in SUVEC during the progress of CSFV infection, the degrees of mTOR phosphorylation in the CSFV -infected SUVEC were examined by Western blotting. SUVEC were infected with CSFV Shimen and CSFV C, and whole-cell lysates were prepared at 0, 24, 48, and 72 h after infection. PBS was incubated as mock infected controls. As displayed in Fig. 1A, infection with CSFV Shimen led to progressive accumulation of p-mTOR signals over time, and the maximal induction was found at 72 h postinfection. In contrast, the increased levels of mTOR phosphorylation were not parallel with the infection of CSFV C in SUVEC (Fig. 8A). The protein levels of mTOR total amounts remained unchanged in the CSFV Shimen or C-infected SUVEC at various time points while *β*-actin was comparable in each sample as a loading control.

To further determine activated mTOR quantitatively in the infected cells, we used ELISA assays to investigate the levels of ability of mTOR phosphorylation at the indicated time points after CSFV infection. As shown by Fig. 8B, there was a time-dependent increase in the mTOR phosphorylation in the CSFV Shimen-infected cells. At 72 h after infection, the activations of phosphorylated mTOR showed approximately 2.7-fold increases versus those in the 0 h cells. Similar results on the kinetics of mTOR accumulation were not found in the CSFV C-infected cells. These data indicate that CSFV Shimen infection induces the activation of mTOR signaling pathway.

## Discussion

CSF is one of the most severe diseases to affect pigs worldwide and has massive economic consequences (Moennig, Floegel-Niesmann & Greiser-Wilke, 2003). With regard to its causative pathogen, the mechanism of its proliferation by utilizing the host cell is still obscure. The present study indicates that the host transcriptome undergoes considerable changes in response to viral infection. Gene regulation is an essential process in the development and maintenance of a healthy body (Way et al., 2014). The regulation of gene expression allows a cell to express specific proteins as and when needed to adapt, to trigger developmental pathways, and respond to viral stimuli (Corada, Morini & Dejana, 2014). CSFV Shimen strains cause acute CSF and induce fatal damage within a short period. Therefore, determining the host cell response is helpful for understanding the pathogenesis of CSF.

Although high-throughput sequencing can provide a large amount of information and a comprehensive view of transcription during viral infection and the cellular response in terms of gene regulation, it increases the difficulty in rapidly and accurately identifying pathogenic regulation-related genes, as the volume of data generated is very large (Georgiou et al., 2014). Evaluating the effects of introducing CSFV C and CSFV Shimen strains into SUVEC as a comparative study aids in understanding the gene-regulatory responses of the host cell that are specifically aimed at controlling infection by pathogenic viruses. Being the attenuated strain used in swine fever vaccine, infection with CSFV C strain does not develop into acute inflammation, haemorrhage, necrosis, or any of the typical lesions of acute swine fever, which are caused by the CSFV Shimen strain; hence, differential response information for the host cell can be filtered to accurately clarify the pathogenic mechanisms of virulent strains.

The present study strongly suggests that RNA splicing regulation has a direct role in disease. Based on pathway and GO analysis, strong links have been established between altered expressions of specific splicing factors, aberrant splicing signalling pathways, and induced signalling pathways that are relevant to transformation or malignancy of cells in normal life activities. Because of the different effects of the CSFV Shimen and C strains, significant differences were observed in the regulation of spliceosome, ribosome, and proteasome pathways, cell cycle, ubiquitin mediated proteolysis, and Wnt signalling pathways and in other series of important signalling pathways in the host cell. The virus altered the homeostasis of gene regulation in the host cell, where the host cell also actively prepares to deal with viral infections of different virulence.

Cellular functions rely extensively on various protein coding (Breker & Schuldiner, 2014). When mRNAs translate into proteins, gene expression is regulated at multiple levels and translational control is critical for gene regulation during development, differentiation, and aging in mammalian systems (Morris & Mattick, 2014; Sonenberg & Hinnebusch, 2009). Alternative splicing offers an exquisite capacity for cells to modify their transcriptome and proteome in response to this regulation (Cooper, Wan & Dreyfuss, 2009). Besides, alternative splicing can regulate the normal function of cells in cell type-, developmental stage-, or signal-dependent patterns (Fu & Jr, 2014). Importantly, recent studies also show that this elaborate scheme shifts the balance of gene expression control as these have emerged in a large number of human diseases resulting from mutations or deregulation of the splicing process (Braunschweig et al., 2013; Wang & Cooper, 2007; Cieply & Carstens, 2015 ). However, disruption of splicing has not been long thought of as a possible mechanism of virus infective disease because of limitations in research techniques.

Based on a tag-based novel high-throughput transcriptome deep sequencing method, our computational analyses of DGE findings suggest that the severity of CSF caused by the virus is attributed to the virus-mediated changes in the characteristics of cell responses at the level of RNA splicing, resulting in a deviation from normal cell characteristics, which is likely to be the fundamental reason why the CSFV Shimen strain contributes to the complete pathogenic host infection and clinical pathology.

First, infection with the CSFV Shimen strain could cause abnormal regulation at the mRNA level by changing the RNA splicing mechanism. Altering RNA transcription further brings about changes in protein synthesis in the ribosome and endoplasmic reticulum, while the normal ribosome functions have a close relationship with spliceosome regulation as an important organelle in protein synthesis (Xiong et al., 2015; Wahl, Will & Hrmann, 2009 ). The CSFV Shimen strain induces splicing regulation in the host, which inevitably reflects on protein synthesis, and different splice forms would result in various results.

In view of the present analysing from a computational study, we further evaluated key gene expression induced by CSFV Shimen or CSFV C. The mTOR signaling pathway is a central regulator of cell survival and growth (Budanov & Karin, 2008). It has been shown that Hepatitis C virus, with CSFV belong to members of the Flaviviridae family, ultimately dependent on the host cell for their replication via activating mTOR signalling pathway (Mannova & Beretta, 2005). It is understandable that the present study showed phosphorylation of mTOR was increased during CSFV Shimen replication.

**Figure 9:**
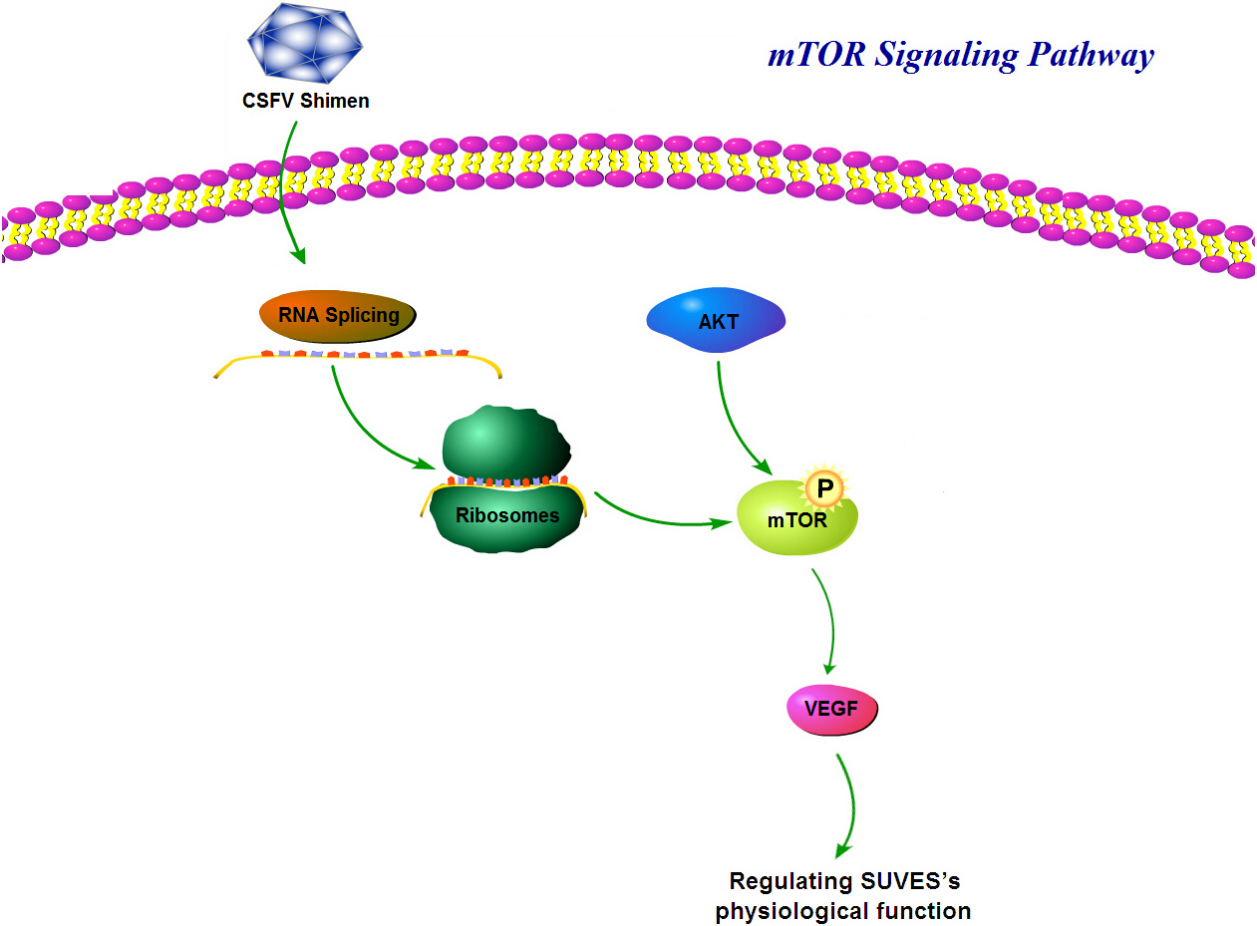
Model of mTOR signalling pathway involved in the infection of CSFV Shimen in SUVEC.

As the upstream of mTOR, Zinzalla et al. (2011) has provided evidence that the ribosome is association with TORC2 activated. As reported in Ning et al. (2013), we first discerned that CSFV Shimen and C strains induced opposite effects on the expression of the VEGF-C gene, a key downstream gene of mTOR that leads to a powerfully increased vascular permeability and is closely related to SUVEC physiological function (Weis & Cheresh, 2014). The present study also provides insight into the mechanism that with increasing proliferation of the CSFV Shimen strain, the balanced regulation of the cell’s normal growth pathways could be damaged, such as mTOR, ultimately leading to clinical disease in pigs (Fig. 9).

In summary, we report the first genome-wide SUVEC transcriptional response to differential CSFV infection by using the Solexa/Illumina DGE system. Our DGE analysis showed massive changes in transcript abundance of SUVEC genes that have been implicated in different CSFV infections. Moreover, mTOR signalling pathway is identified as a significant regulator contributed to impact on SUVEC function by CSFV Shimen. The present study provides a probable mechanism at the mRNA level by which infection with the CSFV Shimen strain disrupts RNA splicing and regulates protein synthesis and degradation to affect normal functioning of SUVEC and finally induces vascular diffuse lesions in the host. The present computational analysis suggests that RNA splicing could be an important contributing factor to infective viral disease, which would provide a new research idea for further study of the CSFV pathogenic mechanism.

##  Supplemental Information

10.7717/peerj.2113/supp-1 Table S1 The key gene products sharing high homology with Sus scrofa in this study This table provides all relevant genes of SUVEC and their transcripts per million mapped reads (TPM) values in the present study. A indicates negative control (mock-infected cells), B indicates CSFV-C group, and D indicates CSFV-Shimen group.

10.7717/peerj.2113/supp-2 Figure S1 Sequencing data saturation in three libraries, control group (mock-infected cells), CSFV-C infected group, and CSFV-Shimen infected group Sequencing data saturation analysis show that the three libraries can be fully saturated with transcripts under different SUVEC samples, and then fewer tags were identified as the number of sequencing tags increased.

10.7717/peerj.2113/supp-3 Figure S2 Differentially expressed genes identified by pairwise comparisons among Control, CSFV-C, and CSFV-Shimen groups The clustered heat map indicates the markedly different overall gene expression patterns among the control, CSFV-C, and CSFV-Shimen groups. The 644, 158, and 677 genes were confirmed to be significantly differentially expressed among the three compared groups (*p* < 0.00015, FDR < 0.001). A indicates negative control (mock-infected cells), B indicates CSFV-C group, and D indicates CSFV-Shimen group.

10.7717/peerj.2113/supp-4 Figure S3 Pathway enrichment analysis for genes in CSFV Shimen-infected SUVEC vs. mock-infected SUVEC The vertical axis denotes the pathway category, and the horizontal axis denotes the negative log values (*p*-values ) for the enriched terms.

10.7717/peerj.2113/supp-5 Figure S4 Pathway enrichment analysis for genes in CSFV C-infected SUVEC vs. mock-infected SUVEC The vertical axis denotes the pathway category, and the horizontal axis denotes the negative log values (*p*-values ) for the enriched terms.

10.7717/peerj.2113/supp-6 Figure S5 Key genes of mTOR signaling pathway in CSFV Shimen libraries vs. CSFV C libraries Red dots represent up-regulated genes in CSFV Shimen libraries vs. CSFV C libraries while green dots represent down-regulated genes in CSFV Shimen libraries vs. CSFV C libraries (*p* < 0.05).

## Additional Information and Declarations

### Data Availability

The following information was supplied regarding data availability: The raw data has been supplied as Supplemental Information.
