# Supplementary figures and images for: Different RNA splicing mechanisms contribute to diverse infective outcome of classical swine fever viruses of differing virulence: insights from the deep sequencing data in swine umbilical vein endothelial cells
Source: PeerJ. 2016 Jun 8;4:e2113. doi: 10.7717/peerj.2113 (PMC4906664; doi:10.7717/peerj.2113)

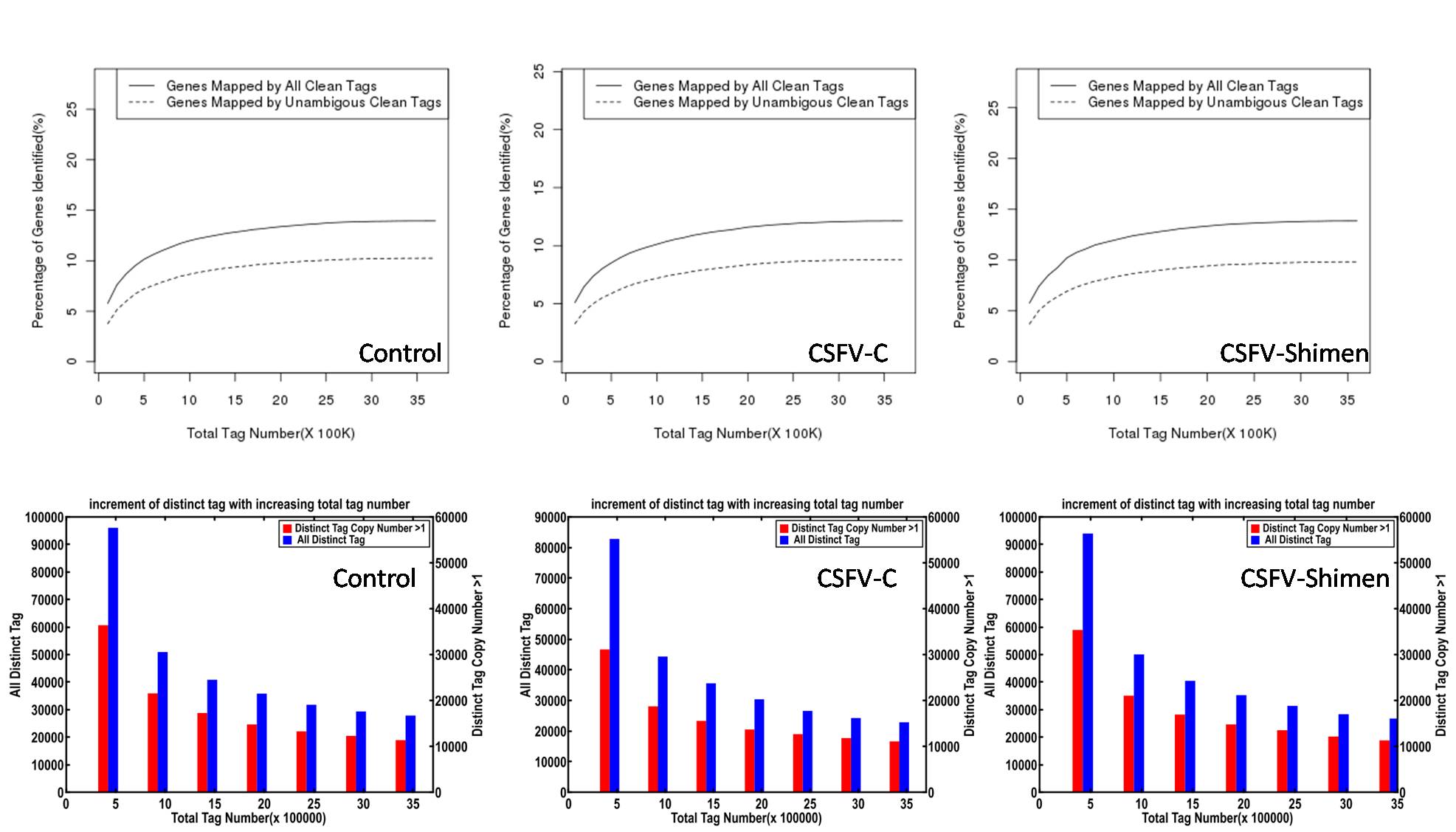

Supplement: Figure S1 — Sequencing data saturation analysis show that the three libraries can be fully saturated with transcripts under different SUVEC samples, and then fewer tags were identified as the number of sequencing tags increased. [file peerj-04-2113-s002.jpg]

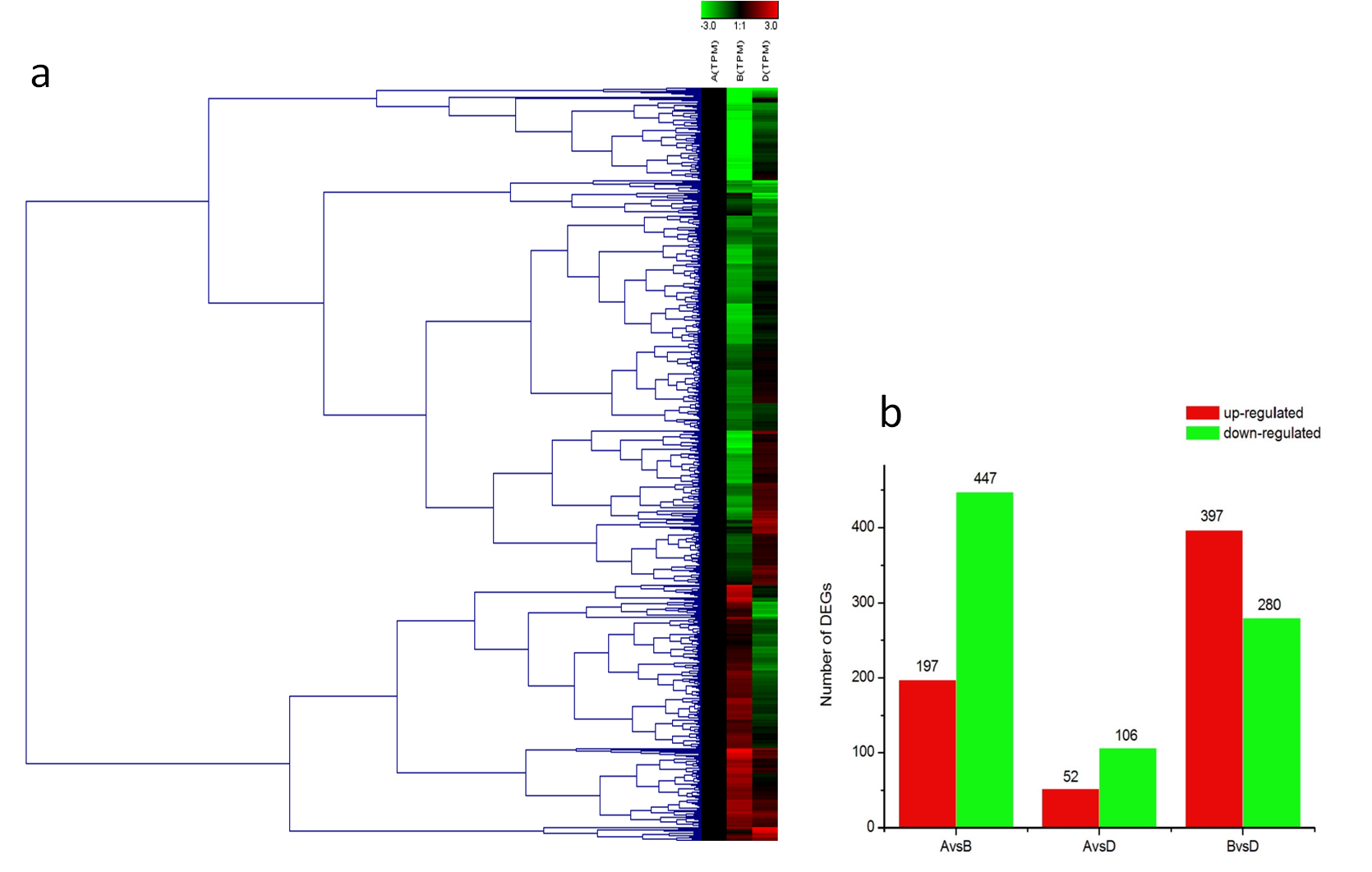

Supplement: Figure S2 — The clustered heat map indicates the markedly different overall gene expression patterns among the control, CSFV-C, and CSFV-Shimen groups. The 644, 158, and 677 genes were confirmed to be significantly differentially expressed among the three compared groups (p < 0.00015, FDR < 0.001). A indicates negative control (mock-infected cells), B indicates CSFV-C group, and D indicates CSFV-Shimen group. [file peerj-04-2113-s003.png]

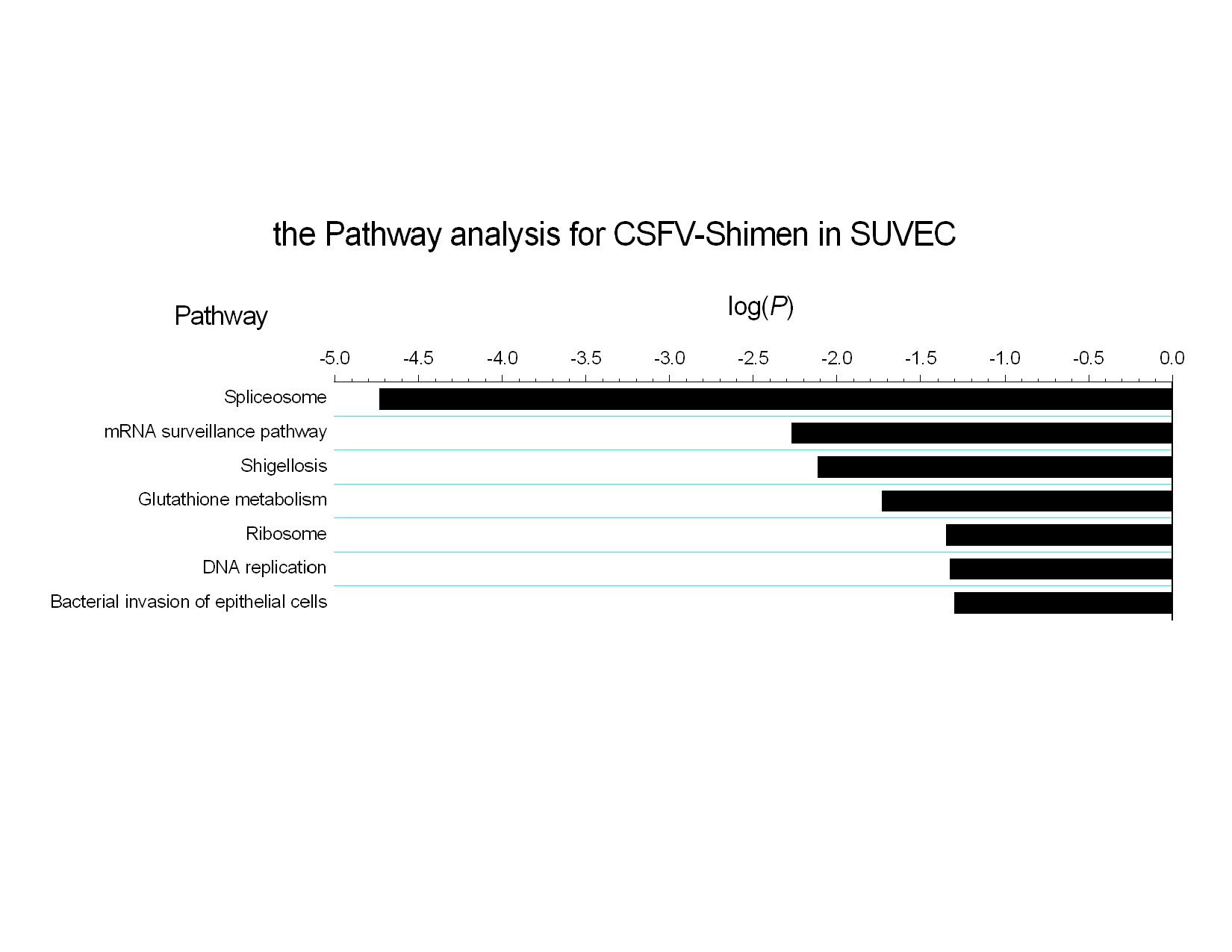

Supplement: Figure S3 — The vertical axis denotes the pathway category, and the horizontal axis denotes the negative log values (p-values ) for the enriched terms. [file peerj-04-2113-s004.jpg]

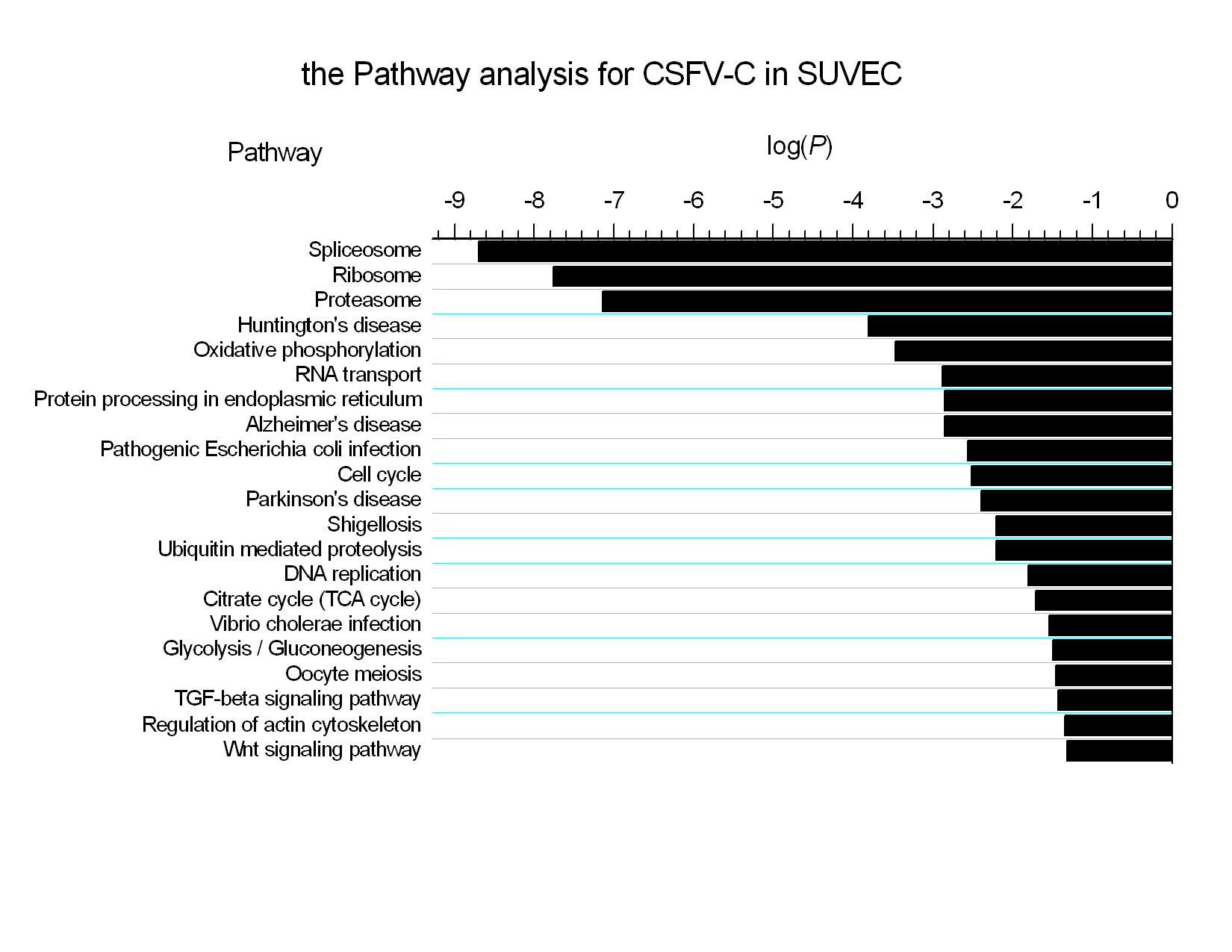

Supplement: Figure S4 — The vertical axis denotes the pathway category, and the horizontal axis denotes the negative log values (p-values ) for the enriched terms. [file peerj-04-2113-s005.jpg]

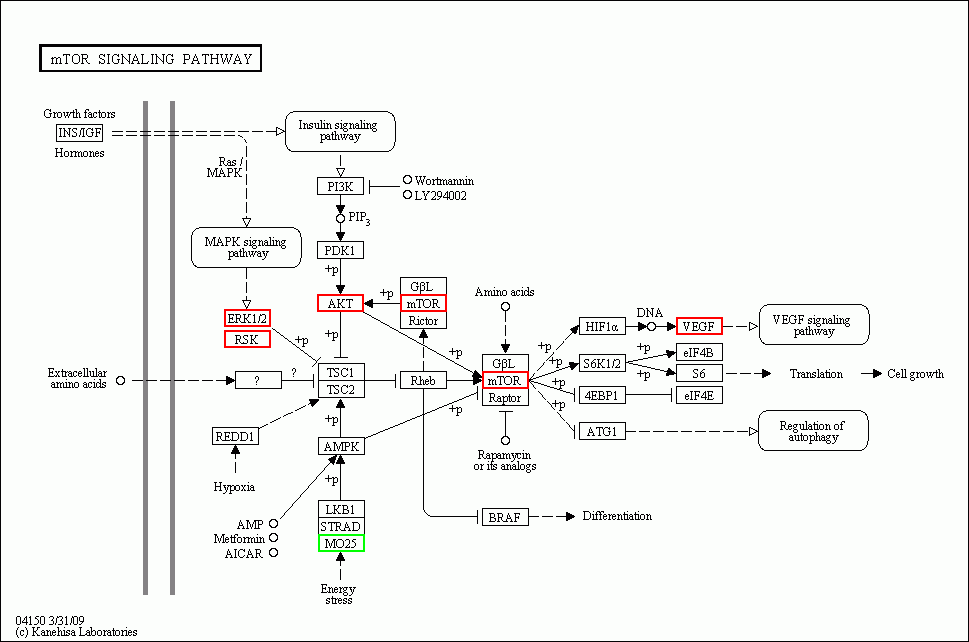

Supplement: Figure S5 — Red dots represent up-regulated genes in CSFV Shimen libraries vs. CSFV C libraries while green dots represent down-regulated genes in CSFV Shimen libraries vs. CSFV C libraries (p < 0.05). [file peerj-04-2113-s006.png]
